# A review of simulation analyses of economics and genetics for the use of in-vitro produced embryos and artificial insemination in dairy herds

**DOI:** 10.1590/1984-3143-AR2020-0020

**Published:** 2020-08-11

**Authors:** Albert De Vries, Karun Kaniyamattam

**Affiliations:** 1 Department of Animal Sciences, University of Florida, Gainesville, FL, USA

**Keywords:** embryo transfer, dairy, profitability, genetic lag

## Abstract

The use of in-vitro produced (IVP) embryo transfer (ET) in dairy herds is growing fast. Much of this growth is on dairy farms where the focus is on milk production and not on selling breeding stock. The value of implementing IVP-ET in a dairy herd arises from a higher genetic merit of the IVP-embryo, but the cost to produce a pregnancy with an IVP embryo is greater than the cost of artificial insemination (AI). The first objective of this study was to review estimates of the net benefit of using IVP-ET over AI in dairy herds using existing literature. Another objective was to show how much IVP-ET use in a herd is optimal. Most of the literature is based on simulation modeling, including our own work that focuses on the dairy industry in the USA. We found that the most profitable use of AI and IVP-ET is often a combination of the two. More IVP-ET should be used when the value of surplus calves is greater and the cost of IVP-ET is lower, among many other factors. In the future, use of IVP-ET will be further improved by more accurately identifying superior donors and recipients, reducing the generation interval, and achieving greater efficiency in embryo production.

## Introduction

Artificial insemination (AI) and in-vitro produced (IVP)-embryos for embryo transfer (ET) are two reproductive technologies that result in genetic gain by propagating offspring from animals with greater genetic merit. The International Embryo Technology Society reported that more than 1 million embryos were produced in-vitro in countries reporting for 2018 (Viana, 2019). Of these, 49% were in North America, 44% in South America, and 6% in Europe. One report states that the combination of IVP-ET with sexed semen and genomic selection is now being successfully and widely used in North America, South America and Europe (Ferré et al., 2020). We will focus here on the USA because of our greater familiarity with its dairy industry.

In the USA, the National Association of Animal Breeders (NAAB, 2020) reported that 22,026,290 units of domestic and imported dairy semen were sold in the U.S. during 2018. Natural service accounts for approximately a quarter of all dairy breedings. Natural service accounts for approximately a quarter of all dairy breedings. The number of transferable IVP embryos of dairy breeds produced in North America during 2018 was 311,458, of which approximately 59% were actually transferred (Viana, 2019). Therefore, approximately 0.5% of dairy breedings were with IVP embryos in the USA during 2018. Use of IVP-ET is growing fast in North America, however. The number of IVP embryos doubled between 2013 and 2017.

Genetic gain has been accelerating since 2010 when genomic testing became widely used to select service sires. The 5-yr moving average rate of genetic gain in predicted transmitting ability (PTA) for the economic selection index Lifetime Net Merit (NM$) is now greater than $70 per year for sires born between 2013 and 2017 (CDCB, 2019). This rate of genetic gain was just $28 per year for sires born between 2003 and 2007. Dairy farms that use only AI make genetic gain in their herds because of genetic gain in marketed AI sires. The Council on Dairy Cattle Breeding (CDCB) data also show that the genetic merit of cows is less than that of service sires. The difference is constant as long as the rate of genetic gain in service sires is constant. Genetic merit of cows lags behind the genetic merit of service sires.

Value of the level of genetic merit in a dairy herd should be based on the difference (genetic lag) in genetic merit between the average cow in the herd and the best available sires (the genetic nucleus; Dechow and Rogers, 2018). This genetic lag is an opportunity cost: each cow consists of “old” sire genetics. For example, when only AI is used and no selection occurs within the herd, the average cow in the herd may be 3.5 yr old. If we assume an annual increase of $50 per year in PTA for NM$, then service sires 3.5 yr ago had a $175 lesser PTA than today’s service sires. The genetic merit of a cow, however, can be thought to consist of 50% her sire + 25% her dam’s sire + 12.5% her grand dam’s sire + 6.25% of her great grand dam’s sire, etc. If the generation interval stays the same between generations, then the genetic lag of the average cow in the herd with the genetic nucleus would be $350 PTA of NM$ (200% × 3.5 yr × $50). This is a doubling (200%) of the genetic lag of the first generation. The genetic lag increases with a greater rate of genetic gain in service sires. If the annual increase in PTA of NM$ is $70 per year, then the genetic lag between the average cow and the genetic nucleus is $490 PTA of NM$ (200% × 3.5 × $70). This math is a simplification of reality, but illustrates the important principle of genetic lag. Results are also herd-dependent.

Selection of superior females in the herd reduces the genetic lag with service sires. For example, use of female sexed semen in younger animals or selection of surplus heifer calves based on genomic test results, produces dairy calves that are on average better than the average unselected dairy calf from the herd. The result is a decrease in the genetic lag with the best available service sires. Use of IVP-ET can greatly decrease this genetic lag as will be illustrated later. Use of technologies such as AI, sexed semen, IVP-ET, and selection of surplus animals all contribute to a reduction in genetic lag.

A greater rate of genetic gain means differences in genetic merit resulting from age become greater. In other words, the difference in genetic merit of the best heifers in the herd compared with the genetic merit of the average cow in the herd is becoming greater when the rate of genetic gain is greater. As a result, capturing and propagating the best genetics in the herd is becoming more valuable.

From the perspective of a typical herd, the genetic merit of available service sires is a given factor that cannot be controlled. When the rate of genetic gain of service sires is constant over time, and the reproduction and selection program for females in the herd are constant over time, it follows that use of technologies like IVP-ET in a herd does not accelerate the rate of genetic gain. They do not increase the annual change as is often thought. It does reduce, however, genetic lag with service sires compared with use of AI.

What is the opportunity cost of genetic lag? Again using simple math, a genetic lag of $350 PTA of NM$ is equivalent to a genetic lag of $700 estimated breeding value (EBV) of NM$ (2 × $350 because EBV = 2 × PTA). We use EBV to express the genetic merit of the female herself, whereas PTA is the genetic merit transmitted to her offspring). The $700 is expressed per lifetime, which is 2.8 lactations, or approximately 3 yr (VanRaden et al., 2018). Thus, the opportunity cost of this genetic lag of $350 PTA of NM$ is $700 ÷ 3 = $233 per cow per year. Using a program that would reduce the genetic lag by $50 is worth approximately 2 × $50 ÷ 3 = $33 per cow per year. One dollar reduction in genetic lag is worth $0.67 per cow per year (simplified). This math does not include any discounting for time value of money, differences in actual lifespan, phenotypic response to selection, and assumes that NM$ is the ideal measure of profitability.

The toolbox of technologies such as AI, sexed semen, beef semen, IVP-ET, genetic evaluations, genomic testing, and fertility programs all affect genetic lag. In addition, these technologies have various direct costs and may affect the phenotypic performance of the herd, such as conception rate. For example, the cost to produce a pregnancy with an IVP embryo is much greater than the cost to produce a pregnancy with AI, but the genetic lag using IVP-ET is smaller. The first objective of this study was to estimate the net benefit of using IVP-ET over AI, which is not immediately clear. Another objective was to show how much IVP-ET use in a herd is optimal, if not to create 100% of pregnancies. The goal of this paper is to provide some insight into these questions.

Transfer of IVP embryos can also improve conception rates in herds with low fertility due to heat stress (Stewart et al., 2011). This type of IVP-ET typically uses oocytes from culled cows. Such oocytes are of average genetic merit because every cow is eventually culled, independent of genetic merit. The average culled cow is approximately 5 years old, so the genetic lag is actually a little greater than the genetic lag with the average cow in the herd. The use of IVP-ET to increase conception rates is not a means to increase genetic merit and we will not further discuss this application here.

## General principles of an IVP-ET program

An IVP-ET program consists of three components: 1) selection of an appropriate ovum pick-up (OPU) protocol; 2) selection of donors; and 3) selection of recipients. Ovum pick up (egg or oocyte collection) is the transvaginal retrieval of oocytes from ovaries of donor females (Hansen, 2017) often after a hormonal treatment. These oocytes are then matured and fertilized in the laboratory resulting in the production of in-vitro embryos. Approximately 1-wk-old embryos are then transferred into nonpregnant recipients and this procedure may result in pregnancies. Typically, donors have reached puberty, but commercial interest in oocyte collection from prepubertal animals is increasing (Moore and Hasler, 2017). Oocytes also can be collected from animals that are up to 4 mo pregnant (Hansen, 2017). The efficiencies of IVP-ET programs vary, but a reasonable number is four transferable embryos per one OPU occurring every 14 d.

Candidate recipients are non-pregnant animals that have a high likelihood of bringing the transferred embryo to term and produce a live calf. Recipients must be approximately on d 7 of their estrous cycle when an embryo is transferred. High fertility, low risk of abortion, and stillbirth are important selection criteria for recipients because of the high cost of IVP embryos. On the other hand, recipients should be animals of relatively lesser genetic merit because they forego the gestation of their own calf. Foregoing the production of their own calf is an opportunity cost. An opportunity cost is the loss of potential gain from other alternatives when one alternative is chosen. Genomic testing also helps more accurately identify recipients.

Donors are those animals eligible for OPU and those that will generate embryos with the greatest genetic merit. Donors should also be free of transmittable disease. To identify such donors, it is useful to rank candidates using a genetic selection index, such as the PTA for NM$. Reliability of PTA based on traditional parent averages (dam and sire of the candidate donor) is low, especially for young animals (≤ 35%; Weigel, 2011). Low reliabilities imply that the difference between PTA (what we know) and true transmitting ability (what it is) of genetic merit of a trait can be large, which might result in selection of donors of low genetic merit. Therefore, genomic testing with much greater reliability (≥ 70%) is routinely used to identify candidate donors and give more certainty that donors with high true transmitting abilities are selected.

**Figure 1:**
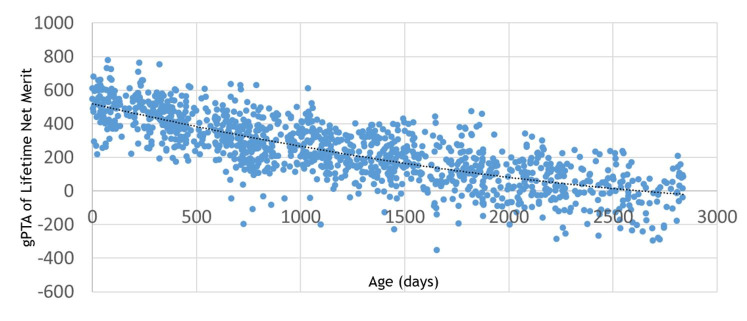
Genomic predicted transmitting abilities (gPTA) of Lifetime Net Merit by age for 1,247 animals at the University of Florida Dairy Unit (2017).

Figure 1 shows genomic PTA of NM$ for 1,247 animals at the University of Florida Dairy Unit. The genetic evaluation was made in 2017. Animals range from a few weeks after birth to more than 2,800 d after birth. The animals were impregnated by conventional and sexed semen, but not IVP-ET. Figure 1 shows a typical distribution of PTA of NM$ as can be found in many herds. Younger animals have greater genomic PTA of NM$ than older cows, but variation exists within the same age. The top young heifers have genomic PTA of close to $800, whereas the average genomic PTA of cows that are 2,500 d old is approximately $0. The genetic trend in these data is approximately $70 PTA per year. This is a greater rate of genetic gain than that in the sires of these females at the time when the females were conceived. The greater rate occurs because of more emphasis on sire selection in the last 4 yr. Therefore, the genetic lag is being reduced. If IVP-ET was to be used in this herd in 2017, 1-yr-old donors would have genomic PTA of approximately $600.

The breeder’s equation (Lush, 1937) predicts change in a trait resulting from selection using a simple statistical model. The four factors that determine genetic change per unit of time are genetic variation, selection intensity, accuracy of selection, and generation interval. An IVP-ET program has a high selection intensity because a small number of genetically superior animals provide many calves for the next generation. It also has a short generation interval because donors are typically young (heifers). Use of genomic testing for both the selection of donors and recipients increases the accuracy (square root of reliability).

In vitro-produced embryos for ET allows for rapid multiplication of the best genetics in the herd, but is also more expensive than AI. There is often an economically optimal amount of IVP-ET to be used, depending on, for example, the value of the calves, cost of the IVP-ET procedure, accuracy of identifying the best dams, and alternative options such as sexed and beef semen. Only a few studies are available that looked at the economics of the use of IVP-ET in dairy herds.

### Economics and Genetic Lag of IVP-ET vs. AI Programs

Ribeiro et al. (2012) calculated a difference in the cost of a female pregnancy to be $329 more for IVP-ET than for AI using sexed semen. This study did not include the value of differences in genetic merit, however. In Denmark, Thomasen et al. (2016) reported that the greatest increase in economic value of genetic gain in a closed population was obtained when juvenile IVP-ET was used along with genomic selection in the bull-dam part of the population. Combining IVP-ET with genomic testing was profitable in almost all evaluated scenarios when the cost of producing a calf (future sire) by IVP-ET ranged from $500 to $1,500. This study therefore looked at the whole population, including the production of service sires. These authors did not study the cost of IVP-ET to improve the female performance in a closed herd. Recently, Sanches et al. (2019) concluded that IVF is becoming an economically viable practice after they reviewed the current use of IVF by large-scale dairy programs.

Several years ago, we built and validated a detailed simulation model that mimics the genetic, technical, and financial performance of a dairy herd over time (Kaniyamattam et al., 2016). The purpose was to investigate how a herd would respond over time to the use of various assisted reproductive technologies such as AI and IVP-ET and genetic selection strategies. We wanted to do this as realistically as possible.

In our model, a dairy herd consisted of individual cows and heifers. Each animal had 12 genetically correlated traits that were present in the 2014 NM$ index, such as milk yield, daughter pregnancy rate (DPR), and productive life. An animal’s performance (milk yield, fertility, risk of forced culling etc.) was the result of her true breeding value (TBV) for each trait, and permanent and environmental effects. Animals also had EBV that were correlated with the TBV for each trait, depending on the reliabilities of the EBV.

Service sires were not part of the herd and followed a genetic trend of $76 PTA of NM$ per year. Therefore, matings with eligible heifers and cows resulted in calves that had PTA depending on those of the dam and the sire and Mendelian sampling (random variation). Heifer calves that were raised likely became cows. Cows already in the herd had a daily risk of culling. Over time, the herd improved genetically as matings with genetically improved sires produced superior dairy calves. The herd, consisting of individual animals, was followed daily and technical results (such as conception rate, milk yield, average TBV, etc.) and financial results (such as milk sales, profitability) were collected for 20 yr into future?

The following general settings were used to study the economics and genetic performance of various AI and IVP-ET strategies: Annual cow cull rate was set at 34% and the herd had 1,000 milking cows. All dairy heifer calves were genomically tested, which gave high reliabilities and therefore high correlations between EBV and TBV. When more dairy heifer calves were born than were needed to replace culled cows, young heifers were ranked based on the EBV of the trait of interest (often NM$) and heifers with the least desirable EBV were sold. Consequently, retained dairy heifers had more desirable TBV on average than unselected dairy heifer calves (similar to analytic results in Weigel et al., 2012) and the genetic lag with the service sires was decreased. This also resulted in greater profitability.

The herd started with using only AI for the first 5 yr. The first two inseminations in the top 50% of heifers were done with sexed semen. All other inseminations were done with conventional semen. After 5 yr, the IVP-ET program was implemented (Kaniyamattam et al., 2017, 2018) and all or some pregnancies were made with IVP embryos. Next, the herd was followed for another 15 yr.

The performance of an IVP-ET system depends on many factors. We assumed that 4.25 transferable embryos were produced per OPU, independent of the age of the donor. Donors for OPU were selected based on rankings for the desirable EBV (e.g., high NM$). The time between OPU of the same donor was 2 wk. Heifer donors could be collected for a maximum of 4 times between 11 mo of age and start of the breeding period. Once a heifer was confirmed pregnant (from AI), she was eligible for 3 more collections. Cows were eligible for a maximum of five collections. Embryos harvested at d 7 after conception were transferred to recipients on d 6, 7 or 8 of the estrous cycle. Recipients were selected based on reverse ranking for the trait of interest (e.g., low NM$), so that the lowest ranked animals had the first chance to receive a randomly chosen IVP embryo.

We assumed that the conception rates was similar for AI and IVP-ET. Conception rates depended on TBV and environmental effects for the traits, DPR and cow conception rate, as well as parity and breeding number. Risk of abortion and stillbirth was at least twice as high in calves made by IVP as from AI. A recent review on post-transfer consequences of IVP embryos in cattle revealed lower conception rates compared with AI (Ealy et al., 2019).

## First study: exclusive Use of IVP-ET or AI

In the first study (Kaniyamattam et al., 2017), we compared four scenarios with exclusive AI use with four scenarios with exclusive IVP-ET use (100% of pregnancies from IVP-ET). Selections of donors and surplus heifer calves were based on one of four selection criteria: EBV of either milk yield, DPR, or NM$, or random selection. Both AI and IVP-ET scenarios produced surplus dairy heifer calves. The lowest ranking surplus calves based on EBV were sold after genomic testing at an age of approximately 3.5 mo. Surplus calves were either sold at $500 each (3 to 4 mo old), or in case of IVP calves at a higher price that included a premium based on the EBV of NM$. The idea here was that surplus IVP calves had greater genetic merit and may be worth more than surplus calves from AI when sold. Cost of production and transfer of one IVP embryo was set at $165. For the IVP-ET scenarios, the top 2% of females were selected as donors. Half of the donors produced oocytes in 1 wk, whereas the other half was not involved in oocyte collection that week. Oocytes were fertilized with female sexed semen.

**Figure 2:**
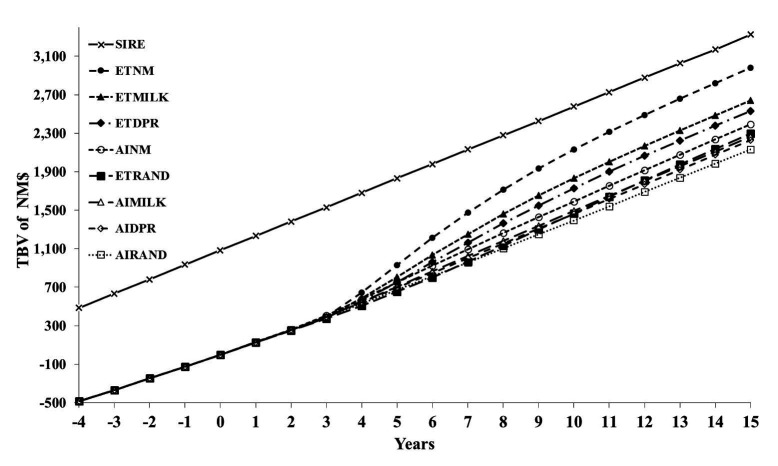
Average true breeding values (TBV) of Lifetime Net Merit (NM$) in sires (SIRE) and cows from year -4 to +15 for 8 scenarios. Name of the scenarios for cows: AI = exclusive artificial insemination program, ET = exclusive in-vitro produced embryo transfer program. Eligible animals were ranked either randomly (RAND) or based on their estimated breeding value of NM$, milk yield (MILK) or daughter pregnancy rate (DPR; Kaniyamattam et al., 2017). Each scenario for cows is a combination of program (AI or ET) and ranking method (NM, MILK, DPR, RAND).

Figure 2 shows the average TBV (2 × PTA) of NM$ in sires and cows from year -4 to +15 after implementation of the 8 scenarios in year 1. The genetic lag between sires and the average cow in the herd before year 1 was approximately $500 PTA of NM$ when no selection among females occurred and only AI was used. The genetic lag started to decrease after year 3 when the first cows started to produce that were conceived after selection criteria were implemented.

The scenarios using IVP-ET and selection of females based on NM$, milk, and DPR all reduced the genetic lag more than the scenario based on AI with selection on NM$. The IVP-ET scenario based on NM$ reduced the genetic lag to $150 PTA of NM$ (= $300 TBV in Figure 2). This constant genetic lag with the service sires was reached approximately in year 13 after the first use of IVP-ET. Thus, from year 3 to year 13 the genetic gain in the females was greater than that in the service sires, but this was the result of moving from the old genetic lag of $500 PTA of NM$ to the new genetic lag of $150 PTA of NM$. In year 15, the AI scenarios produced approximately 30% surplus dairy heifer calves and the IVP-ET scenarios approximately 54% surplus after years of genetic improvement in reproductive traits. This was only 8% in year 0.

**Figure 3:**
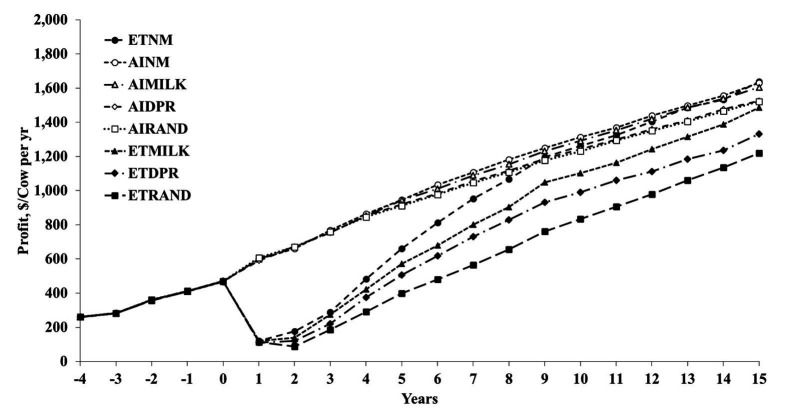
Profit per cow per year from year -4 to +15 for 8 scenarios. Premium pricing of surplus heifer calves is assumed. Name of the scenarios: AI = exclusive artificial insemination program, ET = exclusive in-vitro produced embryo transfer (IVP-ET) program. Eligible animals were ranked either randomly (RAND) or based on their estimated breeding value of Lifetime Net Merit (NM$), milk yield (MILK) or daughter pregnancy rate (DPR). The profit of the 4 IVP-ET scenarios decreases rapidly after the start of the IVP-ET program in year 1 because the embryo transfer cost are greater than the AI cost (Kaniyamattam et al., 2017). Each scenario for cows is a combination of program (AI or ET) and ranking method (NM, MILK, DPR, RAND).

Figure 3 shows profit per cow per year. Change in profitability over time is the combined result of increases in genetic merit and cost of implementing the IVP program from year 1 on. We assumed that there is no inflation. Profitability of the IVP-ET scenarios decreased immediately after year 0 because of the high cost of making IVP embryos. The increased genetic merit of these embryos did not start to pay back until these embryos had become cows (and a little bit as better young stock with improved heifer conception rate).

By year 9, the AI and IVP scenarios with selection based on NM$ started to have similar profitability and by year 15 they differed only by $8 per cow per year (Figure 3; advantage IVP scenario) when the greater surplus calf prices for IVP calves were included. In year 15, the break-even price for an IVP embryo was $168 per transfer, so it was very similar to the input price of $165 (Kaniyamattam et al., 2017).

The AI scenarios were more profitable than the IVP-ET scenarios when the surplus calves were sold for the same price, independent of their genetic merit. With selection on NM$, the break-even price for an IVP embryo was $89. This low break-even price is below current market prices for IVP-ET. The advantage of the AI scenario was $185 per cow per year. The 3 other AI scenarios with selection only on milk yield, DPR, or random selection resulted in greater advantages of AI over the IVP scenarios.

The large decrease in profit per cow in year 1 for IVP-ET program was the result of an immediate transition from AI to IVP-ET where costs were assigned as soon as embryos were transferred. A more gradual use of IVP-ET (< 100%) would avoid this large sudden decrease in profitability, but also delay the reduction in genetic lag and delay in future profitability. This first study showed that 100% IVP-ET programs were typically less profitable than 100% AI programs, even though the genetic lag with service sires was much reduced by the IVP-ET programs.

## Second study: mixed use of IVP-ET and AI

In the second study (Kaniyamattam et al., 2018), we varied the fraction of pregnancies made with IVP-ET from 0% to 100% with intervals of approximately 20%. The best amount of IVP-ET could be less than 100% of pregnancies because the donors would be more superior (fewer are needed) and genetically good animals (that are not donors) would carry their own calves instead of carrying slightly superior but much more expensive calves from IVP-ET. In addition, avoiding recipients that have low conception rates after embryo transfer might be beneficial.

**Figure 4:**
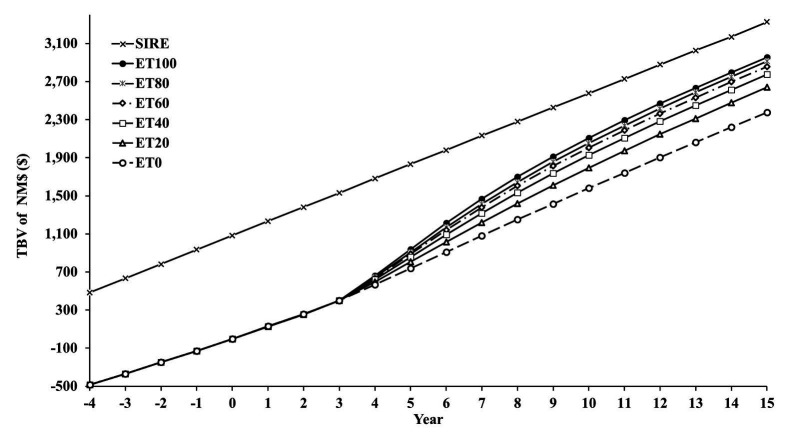
Average true breeding values (TBV) of Lifetime Net Merit (NM$) in sires (SIRE) and cows in  year -4 to +15, in scenarios which used in-vitro produced embryo transfer (IVP-ET) to obtain varying proportions of conceptions from IVP-ET: 0% (ET0), 20% (ET20), 40% (ET40), 60% (ET60), 80% (ET80) and 100% (ET100) (Kaniyamattam et al., 2018).

As expected, Figure 4 shows that the genetic lag with the service sires decreases with greater use of IVP-ET. The rate of decrease in the lag was greatest when IVP-ET use is small. In other words, the more IVP-ET was used, the less the genetic lag changed. It took approximately 10 yr to transition from the old constant genetic lag based on AI only to the new constant genetic lag based on some use of IVP-ET.

**Figure 5:**
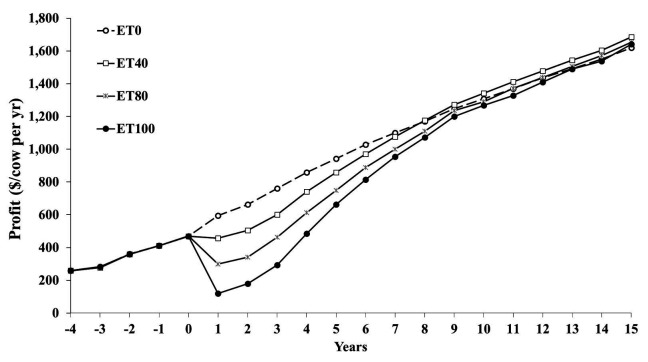
Profit per cow per year in year -4 to +15, in scenarios that used in-vitro produced embryo transfer (IVP-ET) to obtain varying proportions of conceptions from IVP-ET: 0% (ET0), 40% (ET40), 80% (ET80) and 100% (ET100) The cost of the fresh embryo was $165 and the sale price of a 3.5-mo old surplus dairy heifer calf was $500 in addition to a premium price calculated based on the difference of the estimated breeding value of Lifetime Net Merit of sold dairy heifer calves from the IVP-ET scenario compared to the ET0 scenario (Kaniyamattam et al., 2018).

Table 1 shows the results for combinations in: (1) surplus base heifer calf price; (2) premium paid for genetically better surplus heifer calves; (3) IVP-ET price; and (4) the fraction pregnancies from IVP-ET (3 × 2 × 4 × 6 = 144 combinations). As expected, more IVP-ET use was optimal with a greater surplus base heifer calf price, a premium paid for surplus heifer calves, and a lower IVP-ET price. For 6 of the 24 combinations in prices, the 100% IVP-ET program was optimal. All had embryo prices of $100 or less and required a premium paid for genetically better surplus heifer calves. Differences between 0% IVP and 100% IVP could be hundreds of dollars per cow per year when embryo transfer prices were low. When the use of IVP-ET was somewhere in the middle, profitability increased by tens of dollars per cow per year compared with no IVP-ET use or 100% IVP-ET use for the same price assumptions. Figure 5 shows the trend in profitability over time for four scenarios.

**Table 1:** Sensitivity analysis for 6 surplus dairy heifer calf prices and 4 embryo prices and the optimal proportion of conceptions to be achieved by in-vitro produced embryo transfer (IVP-ET) such that profit per cow is maximized (Kaniyamattam et al., 2018).

**Dairy heifer calf sale price** **1**		**Embryo Price**	**ET Conceptions (%)** **2**						**Optimal ET** **4** **^%^**	**Max. Add. Profit** **5** ** ($)**
**Base price**	**Premium**		**0%**	**21%**	**42%**	**63%**	**82%**	**100%**		
			Additional profit per cow in year 15 ($)3							
300	NO	50	0	64	90	80	61	47	46%	91
300	NO	100	0	39	42	7	-36	-75	33%	45
300	NO	150	0	13	-6	-66	-133	-197	19%	14
300	NO	200	0	-12	-55	-139	-231	-319	3%	0
300	YES	50	0	91	155	187	209	241	100%	241
300	YES	100	0	66	107	114	112	119	100%	119
300	YES	150	0	41	58	41	15	-3	42%	58
300	YES	200	0	16	10	-32	-82	-124	28%	18
500	NO	50	35	107	148	150	144	142	69%	158
500	NO	100	35	82	99	77	47	21	41%	99
500	NO	150	35	57	51	4	-50	-101	28%	59
500	NO	200	35	32	3	-69	-147	-223	8%	37
500	YES	50	35	135	213	258	293	337	100%	337
500	YES	100	35	110	164	185	196	215	100%	215
500	YES	150	35	84	116	112	99	93	62%	116
500	YES	200	35	59	67	39	2	-29	36%	69
700	NO	50	70	150	205	221	228	238	84%	238
700	NO	100	70	125	157	187	190	116	73%	199
700	NO	150	70	100	108	75	33	-5	36%	110
700	NO	200	70	75	60	2	-64	-127	12%	77
700	YES	50	70	178	270	328	376	432	100%	432
700	YES	100	70	153	222	255	279	311	100%	311
700	YES	150	70	128	173	182	182	189	79%	191
700	YES	200	70	103	125	109	85	67	25%	129

^1^Base female calf sale prices of $300, $500 or $700 at 105 days of age. The dairy heifer calf rearing cost since birth at 105 days was $375; ^2^Scenario and actual proportion of pregnancies from IVP-ET: ET0 (0%), ET20 (21%), ET40 (42%), ET60 (63%), ET80 (82%), ET100 (100%); ^3^Additional profit per cow in year 15 for varying proportions of conceptions from IVP-ET compared to the scenario with no conceptions from IVP-ET (ET0); ^4^The economically optimal proportion of conceptions obtained from IVP-ET; ^5^The maximum additional profit per cow per year at the optimal proportion of conceptions from IVP-ET compared to the scenario with no conceptions from IVP-ET.

In this second study, the selection of donors was all based on rankings for PTA for NM$ after genomic testing. We did not assume any prior knowledge about the ability of donors to produce transferable embryos. Various factors that determine the production of transferable embryos for an animal are heritable. In one study, heritability estimates for IVP factors in a sample 628 IVP-ET records ranged from 1% to 21%, but were not significantly different from zero (Parker Gaddis et al., 2017). Better understanding of factors that affect the production of transferable embryos should lead to fine-tuning of donor selection.

Further, in this second study we prioritized non-pregnant, non-donor heifers as first eligible to receive IVP embryos. The rationale was that these heifers had greater conception rates than candidate cow recipients and that this was important because of the high IVP-ET prices ($50 to $200). On the other hand, recipient heifers pregnant after IVP-ET have greater opportunity costs of not carrying their own calf compared with recipient cows. Among recipient cows, we gave the highest priority to cows with high PTA for DPR and high PTA for cow conception rate. These cows were expected to have the greatest conception rates, but might not have the lowest PTA for NM$. Again, opportunity cost for the value of their own calf was not considered in selection of cow recipients.

Recipients were selected on the same day the donors were selected. We also ranked candidate recipients independently of their stage in the estrous cycle and looked for estrus daily in the simulation model. If estrus was observed in a selected recipient, the animal was scheduled to receive an IVP embryo on day 6, 7, or 8 after estrus, depending on availability of a fresh embryo. Use of estrus detection instead of estrus synchronization resulted likely in a less than ideal use of candidate recipients, but also at lower direct costs. All eligible animals which were not selected as recipients received AI.

We also assumed that the expected phenotypic performance of calves born from IVP or AI was on average the same if they had the same genetic merit. This may not be the case in practice. For example, in one study, mortality of IVP calves produced by reverse female-sorted semen was greater than in calves produced by AI (Siqueira et al., 2017). Calves born from IVP-ET also have greater risk of large offspring syndrome, which may increase incidences of dystocia and retained placenta (Bonilla et al., 2014). Stillbirths and calf deaths also may increase in IVP calves (Bonilla et al., 2014).

In summary, selection of recipients could be improved by better integration of all factors that determine the profitability of an IVP-ET program. These factors include conception rate, abortion, still birth, value of the IVP-ET calf once born, and the foregone value of the recipient’s own calf. An index that integrates these factors is not too difficult to put together.

Market prices for calves and the value of increased genetic merit fluctuates unpredictably over time. Therefore, the decisions regarding the use of IVP-ET may turn out to be not optimal. A risk analysis with variations in prices over time may result in a policy that is most robust under uncertainty in future prices.

## Outlook

In these two studies by Kaniyamattam et al. (2017, 2018), we assumed that all IVP embryos were made with female sexed semen, and some sexed semen was used in AI for heifers. Consequently, we had a surplus of dairy heifer calves being born and we used genomic testing to help select and sell surplus dairy heifers calves. All dairy bull calves were sold too.

Alternatively, an economically better strategy may be to use AI with beef semen so that crossbred calves are made. Market prices for crossbred calves are approximately $100 greater than those for marketed dairy calves. In contrast, this strategy would lead to fewer surplus dairy heifer calves, and would limit the genetic gain in retained dairy calves because the heifer selection intensity would be lower. A good strategy might involve a combination of IVP-ET, and AI with sexed semen, beef semen, and even conventional semen (Weigel, 2019). We are currently working to identify such promising strategies.

The USDA’s NM$ is a general economic selection index that is useful for a wide range of herds. Other economic selection indexes may be more appropriate in certain markets, such as the Fluid Merit, Cheese Merit and Grazing Merit (VanRaden et al., 2018). A reformulation of the components of the NM$ index using financial investments methods has led to two new economic selection indexes that cause some reranking of service sires (Schmitt et al., 2019). In theory, these new indexes are better at identifying most profitable donors and recipients too.

Further reduction in the generation interval will increase the rate of genetic gain in a nucleus population, for example, in the production of service sires. In-vitro breeding is an emerging technique that greatly reduces the generation interval. It also combines genomic selection with derivation of embryonic stem cells and in-vitro differentiation of germ cells from pluripotent stem cells (Goszczynski et al., 2019). With this technique, the generation interval can be reduced to 3 to 4 mo. This technique may be soon within reach (Goszczynski et al., 2019).

Individual dairy farms that rely on marketed service sires to produce IVP embryos will continue to have a rate of genetic gain that in steady state will be the same as that of the service sires. Improvements in the ranking of donors and recipients, as outlined above, and improved efficiencies and reduced costs will strengthen the economic viability of IVP-ET programs. IVP-ET programs will become more economically competitive with AI programs and eventually they might become clearly more profitable. The best use of IVP-ET on commercial dairy farms remains an interesting puzzle with many variable factors. The modeling approach could also be extended to include in-vivo production of embryos acknowledging differences in costs, fertility, embryonic deaths and production of embryos.
